# Development of humanized mouse models for the preclinical evaluation of NK cell-based therapies

**DOI:** 10.3389/fimmu.2026.1887623

**Published:** 2026-08-17

**Authors:** Pauline Rettman, Samira Benhamouche-Trouillet, Georges A. Azar, Dorothée Bourges, Valeria Fantin, Sukhvinder S. Sidhu, Céline Nicolazzi

**Affiliations:** 1 Sanofi Immuno-Oncology Research, Vitry-sur-Seine, France; 2 Sanofi Immuno-Oncology Research, Cambridge, MA, United States

**Keywords:** huIL-15 transgenic mice, *in vivo* models, monoclonal antibodies, Natural Killer cells, NK cell-based therapies, NK-humanized mouse model

## Abstract

**Introduction:**

Novel immunotherapeutic strategies engaging human Natural Killer (huNK) cells have emerged as a promising treatment paradigm; however, their accurate preclinical evaluation requires reliable mouse models capable of supporting functional huNK cell engraftment.

**Methods:**

We developed and characterized robust *in vivo* NK-humanized mouse models using immunodeficient human Interleukin-15 (huIL-15) transgenic (Tg) mice. *In vitro* amplified huNK cells were used as the source of immune cells. Multiple humanized mouse models were evaluated with optimized protocols. Fcγ receptor knocked-out (FcγR-KO) mice were employed to exclusively model antibody-dependent cellular cytotoxicity (ADCC) potential of huNK cells. A resting period prior to adoptive transfer was also introduced and assessed.

**Results:**

Our NK-humanized mouse models were developed using in vitro amplified huNK cells, which facilitated the production of large number of viable and functional huNK cells and allowed the engraftment of greater cohort of mice. We identified several key parameters improving the long-term persistence and proliferation of functional huNK cells that maintain important functional cell surface markers. FcγR-KO mice exclusively modeled the ADCC potential of huNK cells without the confounding contribution of mouse immune cells. The introduction of a pre-transfer resting period improved tolerability and circumvented potential donor-dependent toxicity issues. Engrafted huNK cells were successfully mobilized in vivo by different NK-based therapeutics, including an engineered IL-2 and an ADCC-enhanced monoclonal antibody.

**Discussion:**

The NK-humanized huIL-15 Tg mouse models developed here provide a versatile and translationally relevant platform for the evaluation and selection of NK cell-based drug candidates, improving clinical translatability and offering a robust framework for preclinical assessment of novel NK cell immunotherapies.

## Introduction

Novel immunotherapies targeting Natural Killer (NK) cells using monoclonal or multi-specific antibodies and immune modulator molecules (1, 2) are under development and there is a pressing need to have suitable preclinical mouse models for their evaluation. Humanized mouse models for the immune system using the adoptive transfer of human peripheral mononuclear blood cells (PBMC) or CD34^+^ hematopoietic stem cells (HSCs) into the first generation of highly immunodeficient mouse strains have been successfully used for the evaluation of T cell-based therapies (3). However, these humanized mouse models have limitations and do not permit optimal development of innate immune cells such as NK, dendritic (DCs) or myeloid cells, due to the poor interspecies cross-reactivity of key factors such as cytokines and growth factors (4).

Interleukin 15 (IL-15) is a critical factor in NK cell homeostasis, proliferation, and survival (5). IL-15 promotes the survival of NK cells in the periphery and is required for the maintenance of mature NK cells *in vivo* (6–9). Several groups have attempted to improve levels of human IL-15 (huIL-15) in humanized mice. Huntington et al. (10) showed that exogenous injections of recombinant huIL-15 and huIL-15Rα expanded human NK (huNK) cells more than huIL-15 administered alone. Chen et al. (11) showed that hydrodynamic tail-vein injections of huIL-15 encoding plasmids led to huIL-15 expression and a subsequent transient elevation of huNK cell numbers for up to 1 month. However, these efforts resulted in only temporary increases in huNK cell proliferation and survival. To address these deficiencies, huIL-15 transgenic (Tg) mice were generated, such as NSG-IL-15 Tg (12) or NOG-IL-15 Tg (13) mice. Alternative strategies have also been envisaged to support huNK cell engraftment such as hIL-7/hIL-15 KI NSG (14), MISTRG (15), NSG-SGM3 (16), NOG-EXL (17) and FLT3L-based models (18, 19). By supporting human myeloid development, these models could allow a more efficient huNK cell development by transpresentation of huIL-15 by human macrophages, as observed in MISTRG mice (20). Humanization with CD34^+^ HSCs in next generation huIL-15 Tg immunodeficient mice also allows the development of functional huNK cells (21), but a drawback of this model is the high cost, need for multiple donors to generate large cohorts of mice for a single study, and the significant length of time required to develop a robust population of huNK cells for the evaluation of NK cell-based therapeutics.

The source of huNK cells should also be considered. While the use of mature human immune cells like PBMCs allows a preeminent engraftment of T cells, it also leads to rapid occurrence of graft versus host disease that could prevent both NK cell establishment and long-term persistence, even in the presence of huIL-15. For these reasons, we decided to utilize *in vitro* amplified human NK cells (22) as the source of immune cells for the development of our NK-humanized models.

While huIL-15 transgenic mice support robust huNK cell engraftment, conventional immunodeficient strains such as NSG and NOG retain a significant innate immune compartment comprising neutrophils, monocytes, and macrophages that express murine Fcγ receptors (FcγR), including the high-affinity muFcγRIV. These murine myeloid cells can be efficiently recruited by human therapeutic monoclonal antibodies and mediate substantial antibody-dependent cellular cytotoxicity (ADCC) independently of human NK cells (23). This confounding contribution of the murine innate compartment complicates the specific attribution of antitumor activity to engrafted human NK cells. To address this limitation and enable mechanistic dissection of human NK cell-mediated ADCC, mouse strains with functional knockout of murine FcγRs have been developed (24, 25), providing a platform to exclusively evaluate the contribution of human NK cells to therapeutic antibody efficacy.

Here, we provide a detailed evaluation of multiple humanized mouse models and optimized protocols to support the long-term maintenance of functional huNK cells for the assessment of NK cell-based therapies.

## Materials and methods

### Cell lines

The chronic myelogenous leukemia cell line K562 (CCL-243, ATCC RRID: CVCL_0004) was engineered to express CD137L-CD86 by Vidar et al (22) and cultured in RPMI-1640 medium (31870-025; Life Technologies) supplemented with 10% heat-inactivated Fetal Calf Serum (FCS) (Eurobio Scientific, CVFSVF06-01), 2 mM L-glutamine (25030-024; Life Technologies), 50µM βmercaptoethanol (31350-010, Gibco), 0.8µg/mL puromycin (P9620; Sigma). Daudi (CCL-213, ATCC, RRID: CVCL_0008) and MM.1R (CRL-2975TM, ATCC, RRID: CVCL_8794) were cultured in RPMI-1640 media + 10% FCS, 2 mM L-glutamine and were used to generate disseminated B cell lymphoma and multiple myeloma models respectively.

Human tumor cell lines Daudi (CCL-213, ATCC) and MM.1R (CRL-2975™, ATCC) were obtained from the American Type Culture Collection (ATCC). Cell lines were authenticated by the supplier using short tandem repeat (STR) profiling prior to distribution. Upon receipt, cell lines were expanded for a limited number of passages and routinely tested for mycoplasma contamination using a PCR-based assay at regular intervals during culture and prior to *in vivo* use. Only mycoplasma-negative cultures were used for experiments. No additional in-house authentication beyond supplier certification was performed.

### huNK cell *in vitro* amplification

Buffy coats from healthy donors were provided by the Etablissement Français du Sang after obtaining written informed consent. huNK cells were purified using the MACSxpress^®^ Whole Blood NK Cell Isolation Kit (130-127-695, Miltenyi Biotec) according to the manufacturer’s instructions. As previously described (22), 5x10^6^ purified NK cells were cocultured with 10^7^ 50-Gy x-irradiated K562-CD137L-CD86 cells, in 20 mL RPMI 10% FCS supplemented with 50 U/ml of IL-15 (570308, BioLegend) and 100 U/ml of IL-21 (571208, BioLegend). The expression level of markers on amplified NK cells was assessed by flow cytometry.

### Animal care

All animal procedures were approved by the Sanofi Animal Care and Use Committee, followed the French and European regulations on care and protection of the Laboratory Animals, and all experiments were performed in institution accredited by the Association for Assessment and Accreditation of Laboratory Animal Care (AAALAC). When anesthesia was required for procedures, mice were anesthetized using isoflurane (inhalation anesthetic) delivered via a MINERVE anesthesia system. Anesthesia was induced at 4% isoflurane and maintained at 2.5% isoflurane in oxygen throughout the procedure. At experimental endpoints or upon reaching humane endpoint criteria, mice were euthanized by cervical dislocation performed by trained personnel in accordance with institutional guidelines and European Directive 2010/63/EU on the protection of animals used for scientific purposes.

### Mouse strains

Female immunodeficient NOG mice expressing huIL-15 cytokine (NOD.Cg-Prkdcscid Il2rgtm1Sug Tg(CMV-IL2/IL15)1-1Jic/JicTac; RRID: IMSR_TAC:13683), also called NOG-IL-15 Tg mice, were purchased from Taconic Biosciences (4623 Lille Skensved, Ejby, Denmark and Wakefield Massachusetts, 01880, United States). Female immunodeficient FcResolv^®^ hIL-15 NOG (NOD.Cg-*Fcgr2btm1Ttk Fcer1gtm1Rav Prkdcscid Il2rgtm1Sug* Tg(CMV-IL2/IL15)1-1Jic/JicTac), presenting a functional knock-out of murine Fcγ receptors, also called FcγR-KO NOG-IL-15 Tg mice were purchased from Taconic Biosciences (Ejby, 4623 Lille Skensved, Denmark). Female immunodeficient NSG mice expressing huIL-15 cytokine (NOD.Cg-*Prkdc^scid^ Il2rg^tm1Wjl^* Tg(IL15)1Sz/SzJ; RRID: IMSR_JAX:030890), also called NSG-IL-15 Tg mice were purchased from Charles River Laboratories (Les Oncins; 69210 Saint Germain Nuelles, France) provided by The Jackson Laboratory (600 Main Street Bar Harbor, ME 04609).

### Mouse models

Immunodeficient huIL-15 Tg mice were irradiated at 0.9 or 1.5 Gy according to the experiment. The irradiation dose was optimized based on strain-specific tolerability during model development. Initial studies in NSG-IL-15 Tg mice established that 1.5 Gy provided optimal myeloid depletion (**Supplementary Figure 1B**) and was well-tolerated. However, when transitioning to NOG-IL-15 Tg mice, we observed that 1.5 Gy resulted in excessive toxicity (pronounced body weight loss, delayed recovery). We therefore reduced the irradiation dose to 0.9 Gy for NOG-IL-15 Tg mice, which provided adequate conditioning while improving tolerability. Both doses (0.9 Gy for NOG-IL-15 Tg and 1.5 Gy for NSG-IL-15 Tg) were subsequently used consistently across both pharmacodynamic and efficacy studies to maintain experimental consistency within each strain. After a 4-days resting period in a quiet environment, irradiated female immunodeficient IL-15 Tg mice were injected intravenously (IV) with 10x10^6^ amplified huNK cells. In Pharmacodynamic studies, mice were euthanized 7, 14, 21 or 28 days after huNK cells transfer to harvest terminal blood, spleen, liver, bone marrow samples followed by flow cytometry analysis from single cell suspensions. In efficacy studies, the activity of either an anti-CD20 (Rituximab Biosimilar, BioXcell, #SIM0008, RRID: AB_2894729) monoclonal antibody (mAb) or anti-CD38 mAb (Daratumumab Biosimilar, BioXcell, #SIM0034, RRID: AB_3696373) was evaluated in a disseminated human B cell lymphoma model, Daudi (CCL-213, ATCC) or multiple myeloma model, MM.1R (CRL-2975™, ATCC), respectively. Either 24h or 6 days after adoptive transfer according to experiments, tumor cells were implanted in the tail vein of NK-humanized mice. Control groups were left untreated. The mAb was administered at 10 mg/kg by intraperitoneal (IP) injections on days 1, 4, and 7 after tumor implantation. Individual mice were weighed daily until the end of the experiment. Survival studies were conducted according to humane criteria and disease-related clinical signs such as limb paralysis, ascites, palpable internal tumor masses, morbidity, or a loss of at least 20% of total body weight loss.

### Age, body weight, randomization, and blinding

Female mice aged 6–12 weeks at the start of the experiments were used, a standard practice in mouse model development in Immuno-Oncology fields to avoid potential confounding effects of male-male aggression, which can complicate group housing and introduce stress-related variability in immune cell engraftment and function. Body weight was measured prior to study initiation and monitored daily; Mice were assigned to treatment or control groups using randomization based on body weight stratification. Due to the nature of the experimental procedures, investigators performing animal handling and treatment administration were not blinded to group allocation. However, data acquisition by flow cytometry and survival endpoints were based on predefined criteria and objective measurements. If blinding was not feasible for specific experimental steps, this was consistent across all groups.

### Inclusion and exclusion criteria

All animals injected with huNK cells and/or tumor cells were included in the analysis. Animals could be excluded from the study only if predefined *a priori* criteria were met, including technical failure during tumor implantation or cell injection, dosing or administration errors, or non–disease-nor-drug related death. Humane endpoint criteria were prospectively defined prior to study initiation and applied uniformly across all experimental groups. Animals reaching humane endpoints were euthanized and considered as events in survival analyses. No animals or data points were excluded from analysis based on treatment response or outcome.

### Staining procedure and flow cytometry

**Figure 1:**
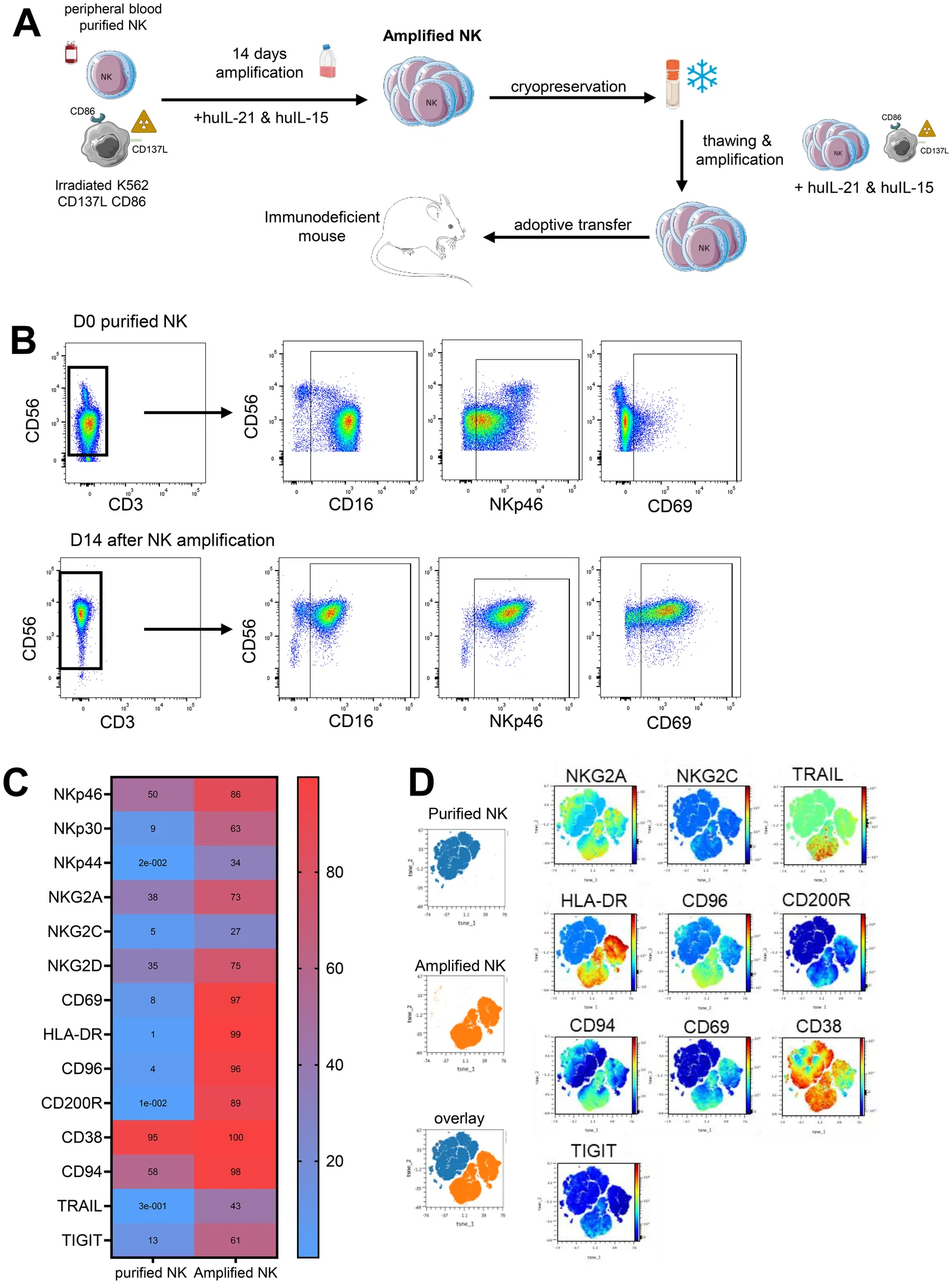
**(A)** *In vitro* amplification process from peripheral blood purified huNK cells in presence of irradiated K562-CD137L-CD86, in media supplemented with huIL-15 and huIL-21. After 14 days, amplified cells (cycle 1) can be cryopreserved to be reamplified for another 14 days (cycle 2), before adoptive transfer. **(B)** Dot plots representing CD16, NKp46 and CD69 expression on day 0 purified vs day 14 amplified CD56^+^CD3^-^ NK cells for one representative donor. **(C)** Heatmap representation of flow cytometry data of purified vs amplified NK cells for 2 representative donors. Red indicates a high proportion of cells expressing the marker, whereas blue indicates a low proportion of cells expressing the marker **(D)** t-SNE plots of NK cell markers from purified vs amplified NK cells. Coloring correlates with the intensity of the expression.

In a 96-well plate, single-cell suspensions prepared from tissues (1x10^6^ per well) were incubated with an antibody mix containing Fc-Blocking buffers and Brillant Stain Buffer (563794, BD) in the dark for 30 min at 4 °C. The Abs are listed in **Supplementary Table 1**. The cells were washed twice in PBS 0.5% FBS 2mM EDTA media. The pellets were resuspended in a 1/1000 dilution of fixable Viability Dye eFluor 780 (L34957, eBiosciences) and incubated for 30 min in the dark, then washed twice before fluorescence acquisition on a BD LSRFortessa x20 cytometer. For blood samples, Precision Count Beads (424902, Biolegend) were added per well for numeration. OneComp eBeads (01-1111-42, eBiosciences) were used for compensation controls. Data were acquired using BD FACSDiva_v9.0 (RRID: SCR_001456) software (or Cytek Aurora for **Figure 1D**) and analyzed with FlowJo_v10 software (RRID: SCR_008520). Unbiased analyses of cellular heterogeneity, e.g. dimensionality reduction and clustering were performed using t-SNE (t-distributed Stochastic Neighbor Embedding) (26).

### Statistical analysis

Detailed information concerning the statistical methods used is provided in the figure legends. Statistical analyses were performed with GraphPad Prism software v.10 (RRID: SCR_002798). Šídák’s multiple comparisons test was used for 2-way-ANOVA comparisons. Kaplan–Meier methods were used for survival analysis. When sample size was sufficiently large, the normality of populations was assessed with the d’Agostino-Pearson omnibus normality test. If the data were not normally distributed, the statistical significance of differences between paired sample populations was determined with the two-sided Wilcoxon signed rank test matched pair. *n* is the number of samples used in the experiments. The means or medians are shown, with or without error bars indicating the standard deviation S.D. Significance is indicated as follows: *P < 0.05; **P < 0.01; ***P < 0.001; ****P < 0.0001.

### Data availability statement

All data supporting the results are available in the main text or the **Supplementary Materials**.

## Results

### Use of *in vitro* amplified huNK cells for adoptive transfer strategies

*In vitro* amplified huNK cells were used as the source of immune cells for the development of our NK-humanized models. HuNK cells were freshly purified from the blood of healthy donors and cultured with irradiated K562-CD137L-CD86 cells in media supplemented with huIL-15 and huIL-21 for 14 days, as previously described (22) (**Figure 1A**). Amplified huNK cells from one donor were cryopreserved then thawed for a second 14-day amplification cycle. From 10x10^6^ thawed cells, 300 to 900x10^6^ NK cells could be obtained (**Supplementary Figure 1A**).

Peripheral blood NK cells are classically divided into 2 subpopulations based on their expression of CD56: CD56^bright^/CD16^-^ and CD56^dim^/CD16^+^ subpopulations (27, 28) as observed for huNK cells just after the purification step (**Figure 1B****),** with a most represented CD56^dim^/CD16^+^ subpopulation. They express low to intermediate levels of natural cytotoxicity receptors and functional NK markers (**Figures 1B, C**). Interestingly, huNK cells amplification induced a homogeneous CD56/CD16-positive NK cell population expressing a variety of functional NK markers. There are higher proportions of cells expressing NKp46, NKp30, NKG2A, NKG2D, CD69, HLA-DR, CD96, CD200R, CD94, TRAIL and TIGIT compared to purified huNK cells, as illustrated in the heatmap (**Figure 1C**) and in t-SNE plots showing that purified and amplified huNK cells form 2 distinct clusters based on the differential expression of NK markers (**Figure 1D**).

### Key parameters for the long-term maintenance of functional huNK cells *in vivo*

To identify the key parameters required for the long-term maintenance of functional huNK cells in mice, we explored the importance of plasmatic huIL-15 levels, preconditioning regimen, source, and dose of huNK cells for engraftment and persistence *in vivo.*

**Figure 2:**
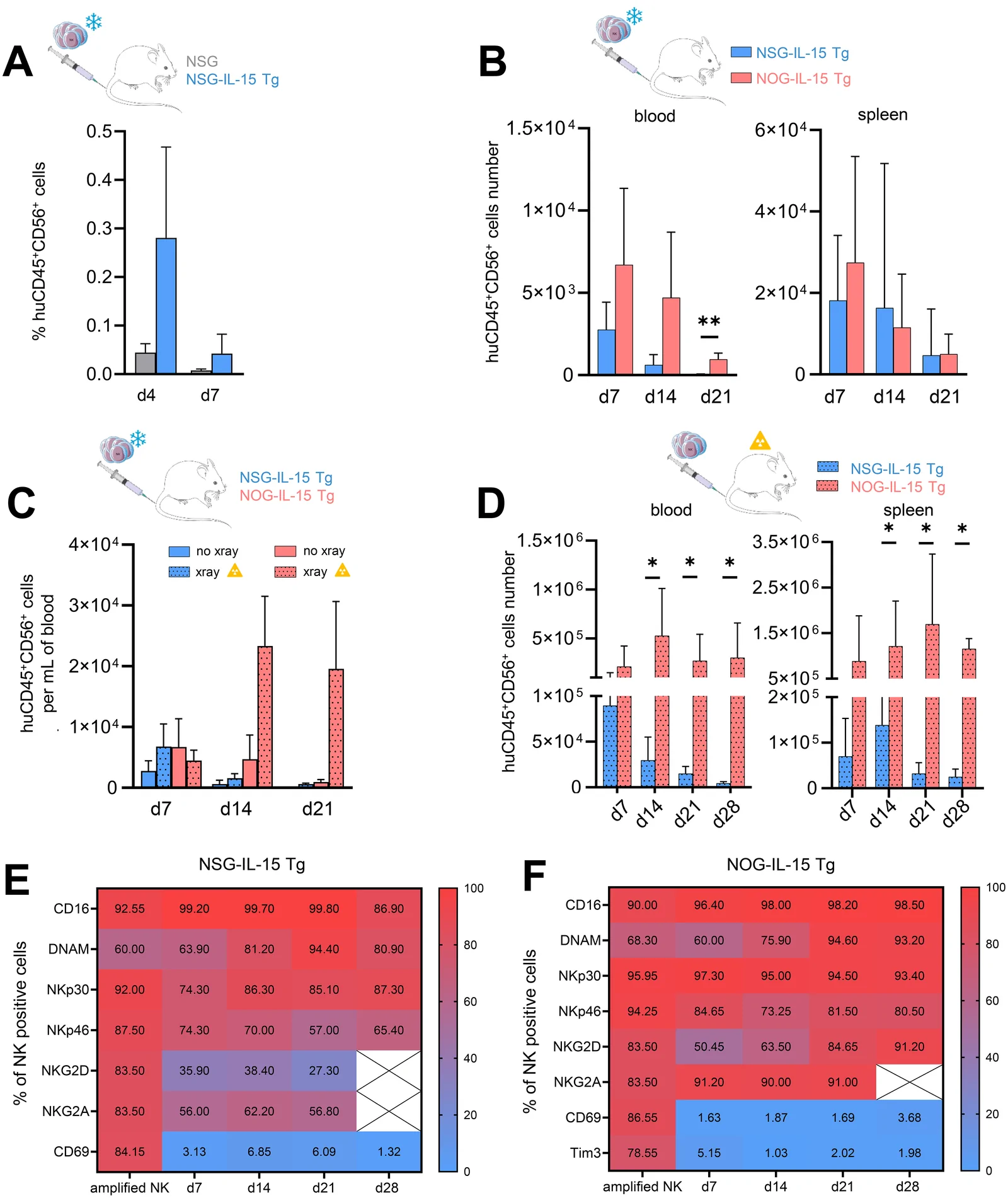
**(A)** Proportion of huCD45^+^ CD56^+^ cells against muCD45^+^ cells in the blood of NSG (gray) and NSG-IL-15 Tg (blue) mice at d4 and d7 after IV injection of reamplified huNK cells, n=3 mice per group. **(B)** Absolute number of huCD45^+^ CD56^+^ cells in the blood and spleen of NSG-IL-15 Tg (blue) and NOG-IL-15 Tg (red) mice at d7, 14 and 21 after IV injection of reamplified huNK cells, n=3–6 mice per group. **(C)** absolute number of huCD45^+^ CD56^+^ cells in the blood of NSG-IL-15 Tg mice (blue, n=3 mice per group) and NOG-IL-15 Tg mice (red, n=6 mice per group) with (dotted bars) or without (plain bars) 1,5 Gy irradiation 4 days before reamplified huNK injection in IV, evaluated at d7, 14 and 21 after transfer. **(D)** Absolute number of huCD45^+^ CD56^+^ cells the blood and spleen of irradiated NSG-IL-15 Tg mice (dotted blue, n=6) and NOG-IL-15 Tg mice (dotted red, n=12) at d7, 14, 21 and 28 after IV injection of freshly amplified huNK cells from several donors. **(E)** Heatmap representing NK marker expression in the blood of NSG-IL-15 Tg mice at d7, 14, 21 and 28 after IV injection of freshly amplified huNK, n=3 mice, for one representative experiment. **(F)** Heatmap representing NK marker expression in the blood of NOG-IL-15 Tg mice at d7, 14, 21 and 28 after IV injection of freshly amplified huNK, data are pooled from 3 independent experiments and NK donors. Statistical significance was assessed by two-way ANOVA with Šídák’s multiple comparisons test and Wilcoxon matched signed rank test. *P < 0.05.

Higher proportions of circulating huNK cells on day 4 and day 7 post engraftment was observed in NSG-huIL-15 Tg mice (10) as compared to NSG mice, highlighting the importance of circulating huIL-15 for huNK cell maintenance *in vivo* (**Figure 2A**). We then compared huNK cells engraftment in transgenic strains displaying different plasmatic levels of huIL-15 (~18 vs 100-150pM in NSG-IL-15 Tg (12) vs NOG-IL-15 Tg (13) mice respectively). To avoid donor-variability, 10x10^6^ huNK cells, from the same donor, reamplified after thawing were injected IV into both mouse strains. Engraftment and maintenance of huNK cells was evaluated by flow cytometry from blood and spleen samples up to 3 weeks after adoptive transfer (**Figure 2B**). In both strains, huNK cells were detectable in similar numbers in the spleen but were more persistent and present in greater numbers in circulation in NOG-IL-15 Tg mice vs NSG-IL-15 Tg mice at day 21.

We next investigated the potential benefit of irradiation to improve huNK engraftment. Complete transient depletion of host neutrophils, monocytes, and macrophages was observed in NSG mice 4 days after irradiation (1.5 Gy) (**Supplementary Figure 1B**). While irradiation of mice did not impact the number of circulating huNK cells in NSG-IL-15 Tg mice, higher and sustained numbers of huNK cells were observed in the blood of irradiated NOG-IL-15 Tg mice up to 3 weeks after transfer (**Figure 2C**). HuNK cells were also maintained in the spleen and liver of irradiated mice in both tested strains (**Supplementary Figures 1C, D****).** Overall, irradiation improved huNK engraftment and maintenance in the blood of NOG-IL-15 Tg mice and in the organs of both strains.

Freshly amplified huNK cells presented greater expansion abilities in all tested donors (**Supplementary Figure 1E**) compared to thawed-reamplified huNK cells (**Supplementary Figure 1A**) and were present in increased numbers in circulation and in spleens of both strains up to 4 weeks after implantation (**Figure 2D**). As previously observed, higher huNK cell numbers were routinely observed in NOG-IL-15 Tg mice, which have higher plasmatic levels of huIL-15, than NSG-IL-15 Tg mice.

Finally, significantly greater numbers of huNK cells were detected in mice implanted with once 10x10^6^ compared to twice 5x10^6^ huNK cells in the blood and spleen of NOG-IL-15 Tg mice (p< 0.05) (**Supplementary Figure 1G**). All further experiments used the optimal dose of 10x10^6^ huNK cells for humanization.

### Inter-donor variability in huNK cell engraftment and persistence

To assess the robustness and reproducibility of the model across different human donors, we analyzed huNK cell kinetics from 4 independent healthy donors whose freshly amplified (cycle 1) huNK cells were transferred into irradiated NOG-IL-15 Tg mice (X-ray, IV injection). Circulating huNK cell numbers were quantified at days 7, 14, 21, and 28 post-transfer (**Supplementary Figure 1F**). While absolute cell numbers varied between donors (ranging from approximately 0.5×10^5^ to 2×10^6^ cells/mL at peak timepoints), all donors supported sustained huNK cell engraftment over the 4-week observation period. Importantly, the temporal kinetics of huNK cell persistence (peak at day 7-14, stable maintenance through day 28) and the expression profile of functional NK markers (CD16, NKp46, NKG2D, NKG2A) remain consistent across all donors (**Figure 2F**). These data demonstrate that while quantitative engraftment levels vary between donors, the qualitative functional characteristics and temporal dynamics of huNK cells in this model are reproducible and donor-independent, supporting the use of this platform for comparative preclinical evaluation of NK cell-based therapeutics.

### Characterization of huNK cells *in vivo*

After a 14-day cycle of *in vitro* amplification, huNK cells are proliferative with ~95% of cells expressing Ki67 (**Supplementary Figure 1H**). *Ex vivo* data from the blood and organs of irradiated NOG IL-15 Tg mice showed that huNK cells maintained over 50% expression of Ki67 at day 7, with decreasing levels over time. These cells also maintained expression of a broad range of functional NK markers, including CD16, DNAM-1, NKp30, NKp46, NKG2D and NKG2A, and decreased expression of activation markers CD69 and Tim-3, suggesting that there may be a decrease in the level of activation observed in huNK cells over time *in vivo* in both huIL-15 Tg mouse strains (**Figures 2E, F**).

### HuNK cells mediate ADCC *in vivo* in huIL-15 Tg mice

**Figure 3:**
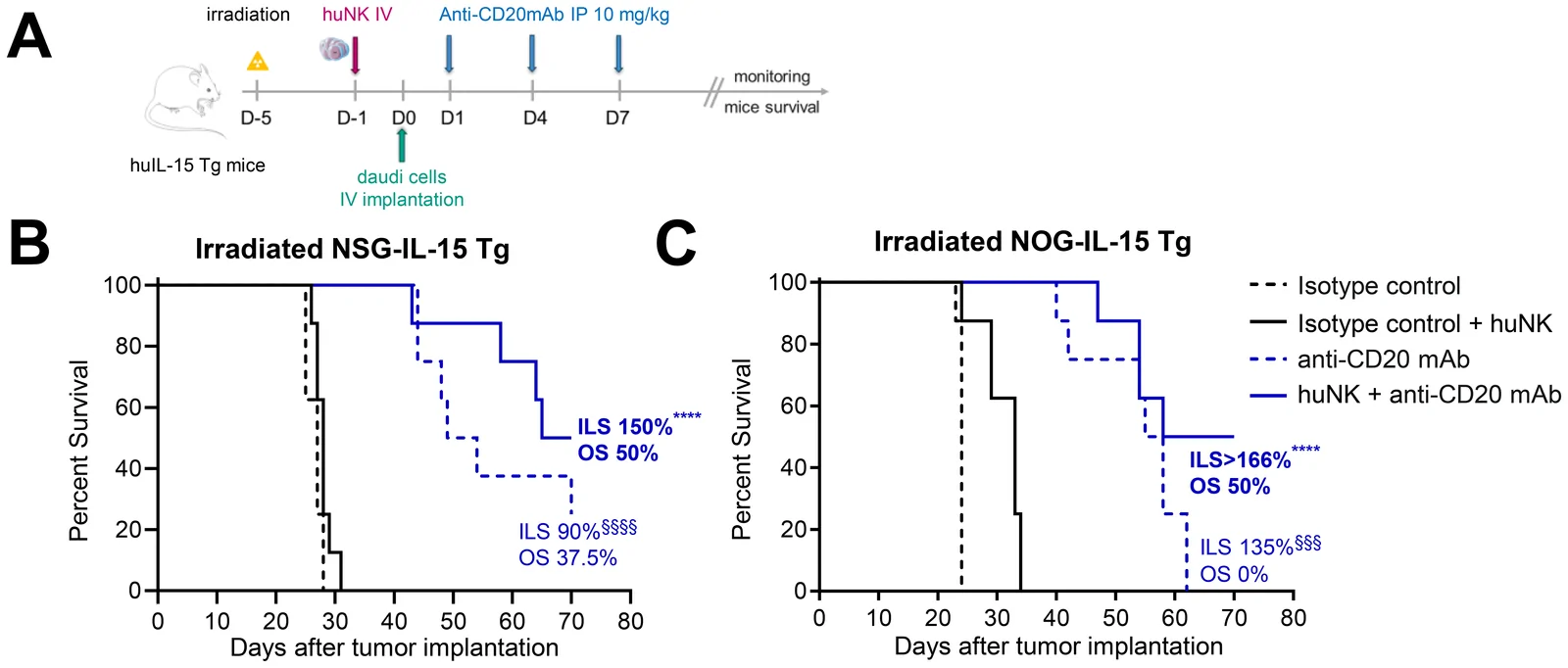
**(A)** Schematic diagram of the experimental setting used in **(B, C)**. IV: intravenous, IP: intraperitoneal. Irradiated NSG-IL-15 Tg **(B)** or NOG-IL-15 Tg **(C)** mice engrafted with reamplified huNK were implanted with Daudi cells in IV and treated every 3 days with anti-CD20 mAb IP at 10 mg/kg alone (dotted blue lines) or in combination with huNK (plain blue line), compared to isotype control (dotted black line) and huNK alone (black line) Data were generated in independent experiments using the same NK donor. Kaplan-Meier survival curves are shown (n=8 mice per group). Statistical significance was assessed by log-rank (Mantel-Cox) test ****p < 0.0001 vs huNK; §§§: p < 0.001; §§§§: p < 0.0001 vs control.

To evaluate the *in vivo* functionality of these NK-humanized mice, efficacy studies were performed with disseminated B-cell lymphoma tumor models (**Figure 3**). In irradiated huIL-15 Tg mice, Daudi cells were implanted one day after IV adoptive transfer of huNK cells, then treated with anti-CD20 mAb every 3 days, for 3 IP administrations at 10mg/kg (**Figure 3A**).

Interestingly, anti-CD20 mAb treatment showed robust antitumor activity with an improved overall survival (OS) and increased lifespan (ILS) in all models, even without NK-humanization (**Figures 3B, C**; **Supplementary Figures 2B, C**). While immunodeficient mice strains such as NSG or NOG do not have functional B, T and NK cells, they still have significant innate immune cell populations such as neutrophils, monocytes, and macrophages (**Supplementary Figure 2A**). These murine myeloid cells express murine Fcγ receptors including muFcγRIV, a high affinity Fcγ receptor allowing them to be efficiently recruited by human mAb and constitute strong immune effector cells (23). Nevertheless, the addition of huNK cells significantly improved the antitumor activity of anti-CD20 mAb with an increased OS and ILS (**Figures 3B, C**; **Supplementary Figure 2C**) in both huIL-15 Tg mouse strains. In contrast, in WT NSG mice, huNK cells cannot survive in the absence of huIL-15 (**Figure 2A**) and do not improve the activity of anti-CD20 mAb (**Supplementary Figure 2B**). To further optimize the model, we evaluated whether the route of administration (IP or IV) of the anti-CD20 mAb could impact recruitment of huNK cells in irradiated NOG-IL-15 Tg mice (**Supplementary Figure 2D**). Interestingly, improved activity of anti-CD20 mAb treatment was observed when treated IV as compared to IP (p=0.0181).

Additional efficacy studies performed in a multiple myeloma model using huNK cells from 6 different donors confirmed the robustness and reproducibility of this model (data not shown). However, variability was noted in the baseline activity of engrafted huNK cells from different donors against the same disseminated MM tumor model, MM1.R (**Supplementary Figure 2E**). Furthermore, engrafted huNK cells alone drove robust antitumor activity in a B-cell non-Hodgkin lymphoma model (WSU-DLCL2) and in an acute monocytic leukemia model (THP1), precluding any assessment of NK-mediated ADCC in these models (**Supplementary Figure 2F**).

### Pre-engraftment conditioning of NK cells to improve tolerability

**Figure 4:**
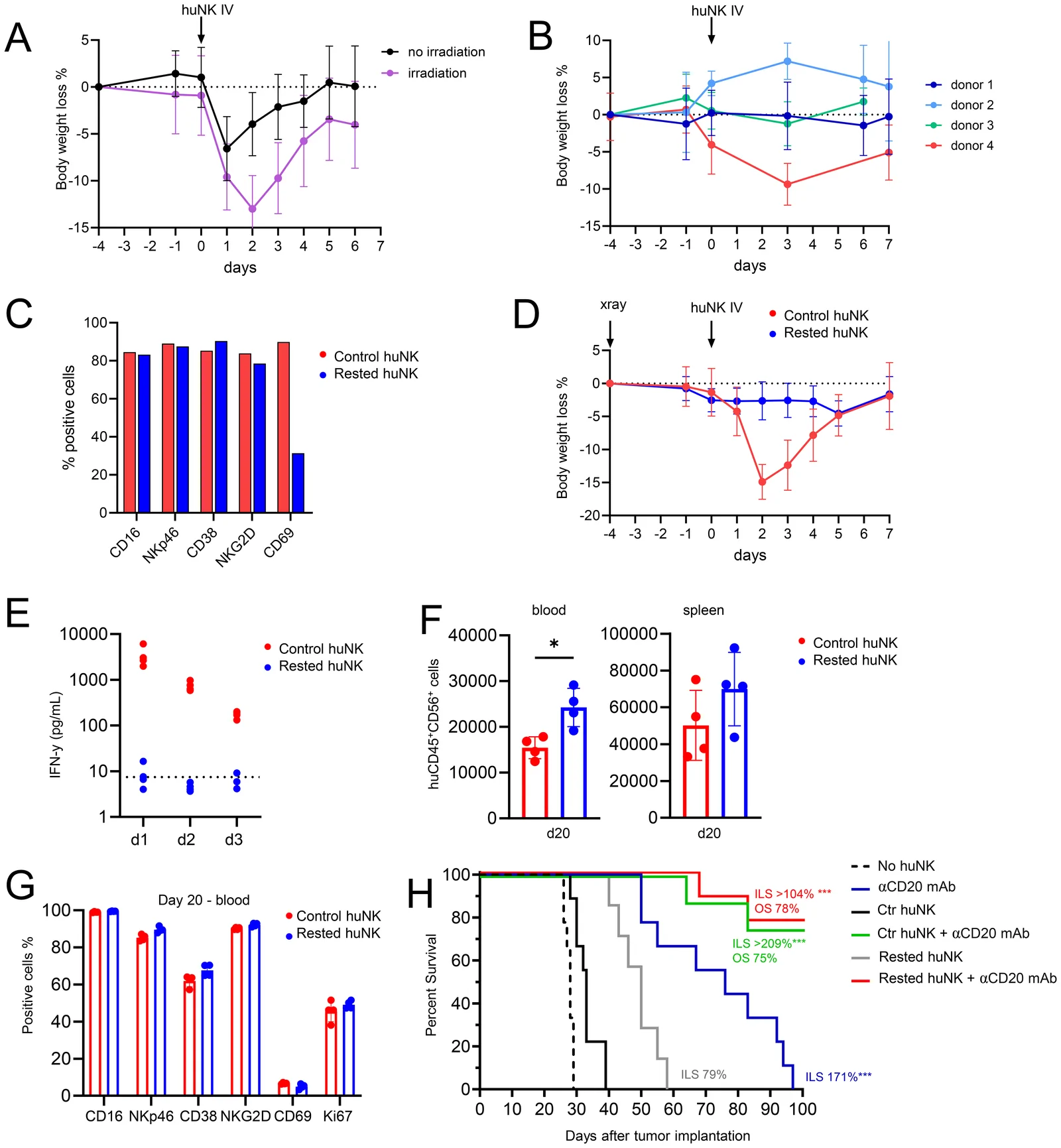
**(A)** Body weight loss curves of NOG-IL-15 Tg mice that were irradiated (purple) or not (black) 4 days before huNK implantation. N = 18 mice per group for one representative huNK donor. **(B)** Body weight loss curves of irradiated NOG-IL-15 Tg mice implanted with amplified huNK cells from 4 different donors, n=8 mice per group. **(C)** Bar graphs representing the level of expression of CD16, NKp46, CD38, NKG2D and CD69 of control (red) or 48h-rested (blue) NK cells for 1 representative donor. **(D)** Body weight loss curves of d-4 irradiated NOG-IL-15 Tg mice implanted at d0 with control (red) or rested (blue) amplified huNK cells (n=4 mice per group). **(E)** Levels of IFN-γ in the plasma of irradiated NOG-IL-15 Tg mice implanted with control (red) or rested (blue) amplified huNK cells (n=4 mice per group). **(F)** Absolute number of huCD45^+^ CD56^+^ cells in the blood and spleen of NOG-IL-15 Tg mice irradiated NOG-IL-15 Tg mice at day 20 after implantation of control (red) or rested (blue) amplified huNK cells (n=4 mice per group) Statistical significance was assessed by unpaired t test *: p < 0.05. **(G)** Level of expression of CD16, NKp46, CD38, NKG2D, CD69 and Ki67 at day 20 after implantation of control (red) or rested (blue) amplified huNK cells (n=4 mice per group). **(H)** irradiated NOG-IL-15 Tg mice engrafted with control (black) or 2 days-rested (gray) amplified huNK were implanted with Daudi cells in IV and treated every 3 days with anti-CD20 mAb IP at 10 mg/kg alone (blue) or in combination with control (green line) or rested huNK (red) compared to control (dotted black line) (n=9 mice per group).; Kaplan-Meier curves were plotted for the analysis of mouse survival. Statistical significance was assessed by log-rank (Mantel-Cox) test ***p < 0.001.

Transient body weight loss was observed 24 to 48h post-huNK cell transfer, which was more pronounced in irradiated animals (**Figure 4A**). Noteworthy, we observed a variability of the tolerability to huNK cells transfer (**Figure 4B**) and some NK donors are not well tolerated, inducing a drastic body weight loss. To mitigate tolerance issues, amplified huNK cells were washed and cultured without cytokines for the final 2–4 days before adoptive transfer. These “rested” huNK cells displayed decreased expression of CD69, but maintained expression of CD16, NKp46, CD38 and NKG2D, as compared to “non-rested” control amplified huNK cells (**Figure 4C**). Rested huNK cells transferred into irradiated NOG-IL-15 Tg mice were well-tolerated and did not induce weight loss as opposed to control huNK cells (**Figure 4D**). Control huNK cells secreted elevated levels of plasmatic IFNγ, while hIFNg was not detected in the plasma of mice grafted with rested huNK cells (**Figure 4E**). Rested huNK cells could be maintained in the blood and spleen in numbers equivalent or superior to those observed with control huNK cells (**Figure 4F**) and maintained expression of functional NK markers (CD16, NKp46, CD38, NKG2D, Ki67) (**Figure 4G**). Furthermore, the resting process did not impact huNK cell functional ADCC abilities as illustrated in an efficacy study in a B cell lymphoma model (**Figure 4H**).

### Evaluating the efficacy of NK-based therapies using NK-humanized mouse models

**Figure 5:**
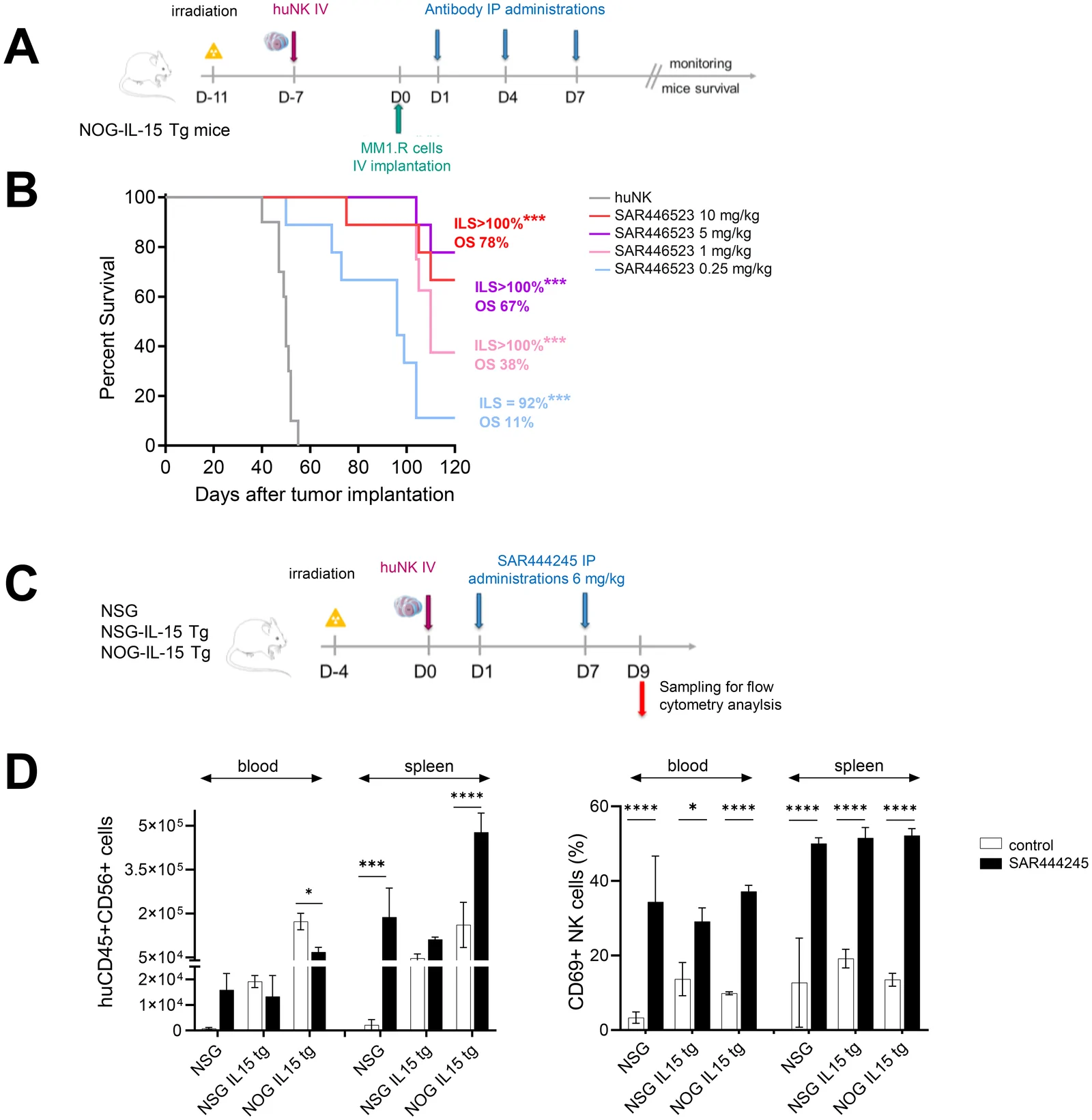
**(A)** Schematic diagram of the experimental setting used in **(B)** Irradiated NOG-IL-15 Tg mice engrafted with huNK were implanted with tumor cells in IV and treated every 3 days with SAR446523 GPRC5D ADCC enhanced antibody at 10 mg/kg (red line), 5 mg/kg (purple line), 1 mg/kg (pink line) or 0.5 mg/kg (blue line) compared to huNK alone (gray line) (n= 9 mice per group). Kaplan-Meier curves were plotted for the analysis of mouse survival. Endpoint significance was calculated in a log-rank test ***: p < 0.001 vs huNK. **(C)** Schematic diagram of the experimental setting used in **(D)** Absolute number of huCD45^+^ CD56^+^ cells (left panel) and proportion of CD69-positive NK cells (right panel) in the blood and spleen of irradiated NSG, NSG-IL-15 Tg or NOG-IL-15 Tg mice engrafted with huNK and treated twice with SAR444245 at 6 mg/kg (black bars) vs untreated (white bars) (n=3 mice per group). Statistical significance was calculated in a Šídák’s multiple comparisons test, *p < 0.05; ***p < 0.001; ****p < 0.0001 vs control.

We next investigated the potential of these models to evaluate the efficacy of other therapeutic antibodies or cytokines. Efficacy of an IgG1-based antibody with an engineered Fc domain targeting GPRC5D, SAR446523, administered at a range of 0.25 to 10mg/kg dose IP was assessed in NK-humanized NOG-IL-15 Tg mice bearing a disseminated multiple myeloma model, MM.1R (29) (**Figures 5A, B**). SAR446523 was active in this model, and we observed dose dependent activity with lower OS for the 0.25 mg/kg and 1 mg/kg doses compared to improved survival with treatment at the higher doses of 5 and 10 mg/kg.

The impact of a non-alpha engineered human IL-2, SAR444245, was evaluated on huNK cell persistence and activation after their implantation in NSG and huIL-15 Tg mouse models (**Figures 5C, D**). SAR444245 sustained huNK cells in the blood and spleen of NSG mice in the absence of circulating huIL-15. In the IL-15 Tg mouse models, huNK cells were observed at similar or higher levels in blood and spleen. However, increased CD69 positive cells were observed after SAR444245 treatment in all tested strains, highlighting that a non-alpha IL-2 can activate NK cells *in vivo*, even in presence of circulating huIL-15. In summary, we showed the ability of huNK cells to be mobilized *in vivo* by different NK-based therapeutics.

### Evaluating NK-mediated ADCC activity without the confounding impact of murine effectors

**Figure 6:**
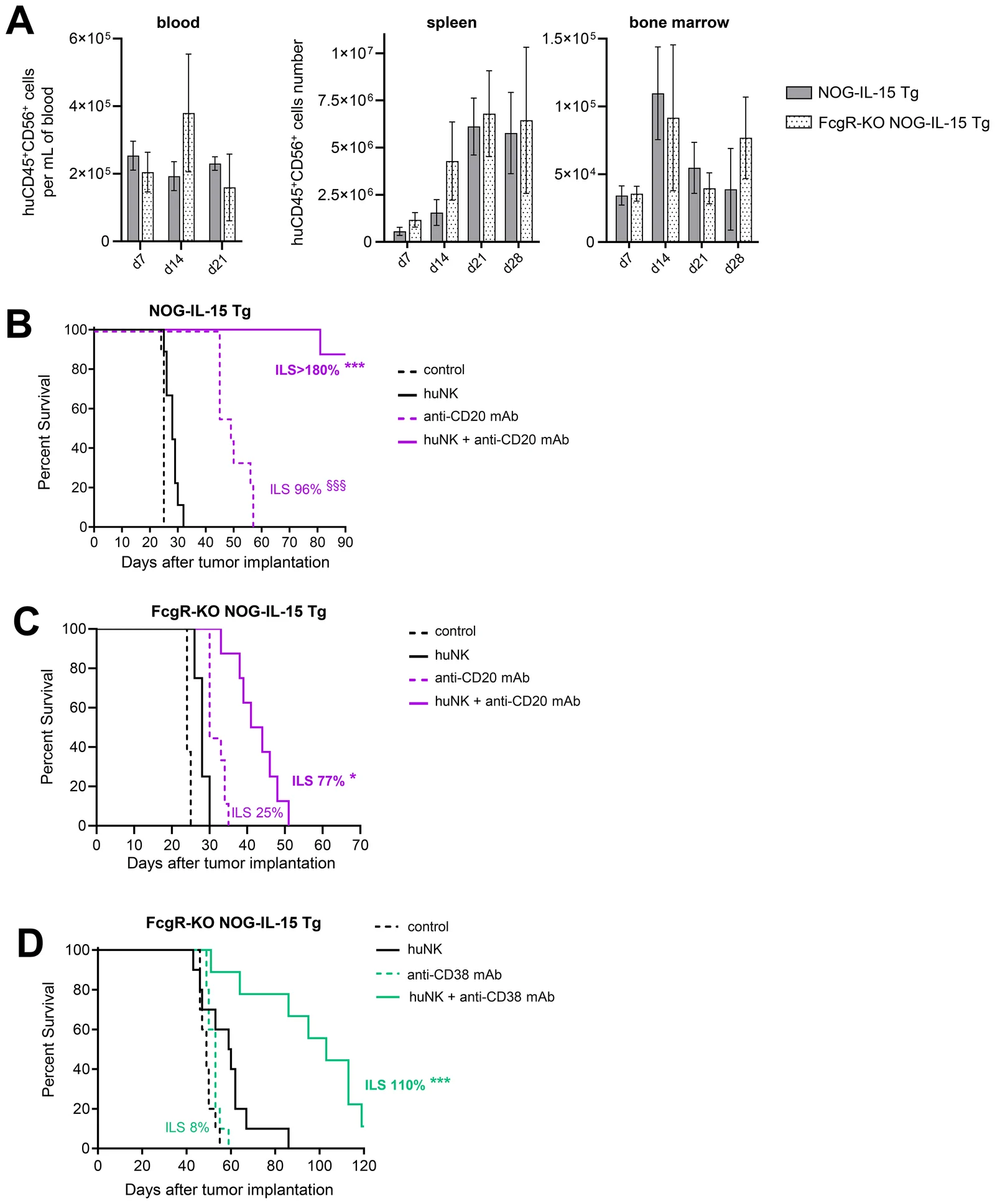
**(A)** Absolute number of huCD45^+^ CD56^+^ cells in the blood, spleen and bone marrow of NOG-IL-15 Tg (gray) and FcγR-KO NOG-IL-15 Tg (dotted white) mice at d7, 14, 21 and 28 after transfer of 10x10^6^ freshly amplified huNK cells in IV (n=4 mice per group). Irradiated NOG-IL-15 Tg **(B)** or FcγR-KO NOG-IL-15 Tg **(C)** mice engrafted with huNK were implanted with Daudi cells in IV and treated every 3 days with anti-CD20 mAb IP at 10 mg/kg alone (dotted purple lines) or in combination with huNK (plain purple line), compared to control (dotted black line) and huNK alone (black line) (n= 9 mice per group). Kaplan-Meier curves were plotted for the analysis of mouse survival. Data was generated in independent experiments using different NK donors. Endpoint significance was calculated in a log-rank test. *p < 0.05; ***p < 0.001 vs huNK; ^§§§^: p < 0.001 vs control. **(D)** Irradiated FcγR-KO NOG-IL-15 Tg mice engrafted with huNK were implanted with MM.1R cells in IV and treated every 3 days with anti-CD38 mAb IP at 10 mg/kg alone (dotted green lines) or in combination with huNK (plain green line), compared to huNK alone (gray line), (n=10 mice per group). Endpoint significance was calculated in a log-rank test ***: p < 0.001. Data was generated in independent experiments using different NK donors.

To minimize the confounding impact of the murine myeloid compartment, we next investigated a NOG-IL-15 Tg mouse strain with a functional knock-out of murine Fcγ receptors (FcγR-KO) (24, 25). Similar number of huNK cells were detected in blood, spleen, and bone marrow at days 7, 14, 21, 28 after adoptive transfer in irradiated FcγR-KO NOG-IL-15 Tg as compared to NOG-IL-15 Tg mice (**Figure 6A****).** HuNK cells implanted in FcγR-KO NOG-IL-15 Tg mice had the same proliferating profile (**Supplementary Figure 2G**) as in NOG-IL-15 Tg (**Supplementary Figure 1G**), with high Ki67 expression at day 7 and a gradual decrease up to day 28. They maintained expression of CD16, NKG2D, NKp46 and did not express CD69 (**Supplementary Figure 2H**), as observed in NOG-IL-15 Tg mice (**Figure 2F**).

*In vivo* efficacy studies were performed in a B-cell lymphoma model treated with an anti-CD20 mAb after huNK adoptive transfer in both models (**Figures 6B, C**). Additive effects of huNK cells to the baseline murine effector cells antitumor activity can be seen for the CD20 Ab with an ILS of 96% without huNK cells and ILS of >180% with huNK cells, in NOG-IL-15 Tg mice (**Figure 6B**). In FcγR-KO mice, we observed no activity of the anti-CD20 mAb alone (ILS of 25%) as murine cells can no longer be recruited, but an improved ILS of 77% in the presence of huNK cells, illustrating their role as principal effector cells mediating antitumor activity in this model (**Figure 6C**). Similarly, in a disseminated multiple myeloma model (MM1.R), we observed that in FcγR-KO NOG huIL-15 Tg mice, anti-CD38 mAb alone showed no activity while a robust antitumor activity is only observed in the presence of huNK cells (ILS 8% vs 110%) (**Figure 6D**).

## Discussion

To evaluate new immunotherapeutic approaches targeting NK cells, reliable mouse models are essential. The adoptive transfer of human PBMCs or CD34^+^ HSCs present significant limitations because of a suboptimal huNK cells maturation and development. Here, we have established robust and well-characterized *in vivo* models allowing the engraftment and long-term maintenance of fully functional huNK cells in immunodeficient mice transgenic for huIL-15. We identified several key parameters that improved huNK cell engraftment and persistence *in vivo* and determined the optimal conditions for establishing a robust model.

HuNK cells used for adoptive transfer strategies were expanded *in vitro* beforehand, resulting in a homogeneous CD56/CD16 double-positive population expressing high levels of huNK cell receptors and we confirmed the maintenance of expression of important functional markers on these cells *in vivo*. While this method facilitated the production of large numbers of viable and functional huNK cells allowing the engraftment of large numbers of mice, one drawback was some donor-dependent tolerability issues post engraftment. We circumvented this issue by introducing a resting period before the adoptive transfer of huNK cells and showed that these cells are still fully viable and functional.

Our comparative analysis revealed superior huNK cell persistence in the peripheral blood of NOG-IL-15 Tg mice compared to NSG-IL-15 Tg mice (**Figures 2B–D**). Plasmatic huIL-15 levels in NSG-IL-15 Tg vs NOG-IL-15 Tg mouse strains are ~18 vs ~100pM respectively. These levels are superior to IL-15 serum levels observed in human (~1 pM). While in both huIL-15 Tg mouse strains, plasmatic huIL-15 levels can sustain huNK cells, the higher level of plasmatic huIL-15 in NOG-IL-15 Tg mice allowed a better huNK cell engraftment and, particularly, long-term maintenance in the blood compartment than in NSG-IL-15 Tg mice. In addition, huNK numbers observed in the peripheral blood of NOG-IL-15 Tg mice are comparable to human (253 NK cells/µL, with an important variability from 82 to 594 cells/µL (30)) and stable over time (for 4 weeks) while similar huNK numbers in the peripheral blood of NSG-IL-15 Tg this huNK level are only observed for 1 week. Of note, huNK cells maintained similar phenotypic profiles (expression of CD16, NKp46, NKG2D, NKG2A) in both strains (**Figures 2E, F**), indicating that the quality of huNK cell maintenance is comparable and that the primary difference is quantitative persistence, consistent with an IL-15 dose effect. Our experimental design does not allow complete dissociation of the IL-15 dose effect from potential strain background effects, as both NSG and NOG parental strains (without IL-15 transgene) are comparably permissive for human immune cell engraftment (3), suggesting that baseline strain differences in the hematopoietic niche are minimal. However, these supraphysiological IL-15 levels represent a necessary compromise inherent to all current huNK cell-humanized mouse models, so for practical purposes NK-humanized NOG-IL-15 Tg mice are the preferred platform for extended pharmacodynamic and efficacy studies requiring sustained NK cell presence beyond at least 4 weeks.

Furthermore, engrafted huNK cells were able to mediate robust antitumor activity with an anti-CD20 mAb in B-cell lymphoma models. We highlighted the value of our NK-humanized mouse models to evaluate a variety of NK-based therapeutics, including an engineered IL-2 and an ADCC-enhanced mAb. However, in 3 other disseminated tumor models, strong NK-mediated activity was observed in absence of treatment, preventing the assessment of NK-mediated ADCC. Understanding what drives this intrinsic susceptibility to NK lysis in these tumor models is a focus of our future studies. Exploring the expression of additional NK markers such as NKG2D, DNAM-1 or Killer-cell Immunoglobulin-like Receptors (KIR) on NK cells and their ligands on tumor cells may provide insight into the ability of direct NK cell tumor killing (31–36).

To better understand the mechanism of action of these NK cell-based therapies and to further discriminate the role of huNK cells versus mouse effector cells, we validated a mouse strain with a functional knock-out of murine Fcγ receptors that prevents the recruitment of mouse effector cells and consequently avoid their confounding impact on antitumor activity. In immunodeficient strains such as NSG and NOG, the innate cell compartment contains a significantly larger population of innate effector cells (macrophages, monocytes, DCs) than its human counterpart (4), which may provide a disproportionate contribution to antitumor response. Therefore, there is an added translational value of dissecting out individual immune compartments. In NK-humanized FcγR-KO NOG-IL-15 Tg mice, we showed that engrafted huNK cells alone, without the intervention of murine effector cells, can drive robust ADCC-driven antitumor activity in the presence of a therapeutic antibody, validating our NK-humanized IL-15 Tg murine models.

A limitation of the models developed with only huNK cell engraftment is that they are not suitable to evaluate the ability of NK cells to orchestrate a complete and durable immune response via crosstalk with other human immune system components such as T cells, myeloid cells, and DCs. For example, NK cells can induce the recruitment of DCs into solid tumors via chemokine ligands that have been correlated with improved OS in patients (37). The crosstalk between NK cells and DCs can also promote DC uptake of tumor antigens in secondary lymph nodes for presentation to and activation of T-cells (38), which facilitate additional antitumor responses in the tumor microenvironment. In the absence of a human hematopoietic system, murine stromal and myeloid cells cannot provide the physiological trans-presentation of IL-15 required for human NK cell homeostasis, necessitating systemic transgenic expression or exogenous supplementation. Alternative models such as MISTRG (15), NOG-EXL (17), and FLT3L-based systems (18) face similar challenges.

In summary, we have developed NK-humanized models with long term persistence of functional huNK cells, that can be used to assess NK cell recruitment (39, 40). These models are ideal for proof-of-concept studies and assess the mechanism of action of NK-based therapeutics. We highlighted the cutting-edge capabilities of the developed NK-humanized mice models, demonstrating their potential for the evaluation of NK-based therapies in hematological cancers (29), and further in solid tumors (41). These new models could also be used to evaluate combinations with standard of care treatments such as anti-CD38 mAbs (42). Other models that were not in scope of this study but could be of interest to explore include human hematopoietic stem cell engrafted NSG-huIL-15 Tg mice (21), that should support more diverse human immune cell populations, including both NK and T cells. However, caution must be taken in how different levels of plasmatic huIL-15 may impact the broader development of the human immune system as there are preliminary reports that NOG-IL-15 Tg mice do not support the engraftment of CD34^+^ HSCs. Other new murine models recently published that could be explored include the new humanized THX mice (43), however, the functionality of NK cells, in addition to their presence in this model needs to be further investigated.

The NK-humanized huIL-15 Tg mouse models developed in this study occupy a specific niche in the spectrum of available humanized mouse platforms. The models are best suited for rank-ordering therapeutic candidates, identifying mechanisms of action, and establishing proof-of-concept for NK cell engagement strategies. To guide appropriate model selection for preclinical studies, we provide the following framework: these models are well-suited for proof-of-concept studies evaluating the *in vivo* activity of NK cell-engaging therapeutics, including ADCC-enhanced monoclonal antibodies, NK cell engagers, and NK-activating cytokines; for mechanistic studies dissecting the specific contribution of human NK cells to therapeutic antibody efficacy, particularly when using the FcγR-KO NOG-IL-15 Tg strain to eliminate confounding murine effector cell activity; for pharmacodynamic evaluation of NK cell persistence, activation, proliferation, and phenotypic modulation in response to therapeutic interventions; for rank-ordering and selection of drug candidates based on comparative NK cell recruitment and antitumor activity in hematological malignancy models; and for rapid turnaround studies requiring large cohorts of mice with functional human NK cells, leveraging the scalability of *in vitro* NK cell expansion (300-900 × 10^6^ cells per amplification cycle, **Supplementary Figure 1A**).

However, they are not well-suited for the evaluation of complete adaptive immune responses requiring functional human T cells, dendritic cells, and antigen presentation pathways. For applications requiring broader human immune system reconstitution, alternative models such as CD34+ HSC-engrafted NSG-IL-15 Tg mice (21), MISTRG mice (15), or the recently described THX mice (43) may be more appropriate, with the caveat that these models require significantly longer lead times (12–16 weeks for HSC engraftment) and higher costs. The choice of model should be guided by the specific biological question, the therapeutic modality under investigation, and the balance between experimental complexity and translational relevance.

Overall, the new models that we have developed will improve selection of drug candidates and clinical translatability. 

## Data Availability

The raw data supporting the conclusions of this article will be made available by the authors, without undue reservation.
