# Supplementary material for: Development of humanized mouse models for the preclinical evaluation of NK cell-based therapies
Source: Front Immunol. 2026 Aug 17;17:1887623. doi: 10.3389/fimmu.2026.1887623 (PMC13535524; doi:10.3389/fimmu.2026.1887623)
Supplement: Supplementary file 1 [file Table1.docx]

Supplementary Material

humanized mouse models to evaluate NK therapies

Supplemental Table 1. mAb used in Flow cytometry

| **Marker** | **Fluorochrome** | **Reference, Supplier** | **clone** | **RRID** |
| --- | --- | --- | --- | --- |
| CD45 | BUV805 | 612891, BD | HI30 | AB_2870179 |
| CD3 | BV510 | 563109, BD | UCHT-1 | AB_2732053 |
| CD56 | BV421 | 562751, BD | NCAM16 | AB_2732054 |
| CD16 | BUV496 | 612944, BD | 3G8 | AB_2870224 |
| CD335/NKp46 | APC | 130-112-122, Miltenyi | REA808 | AB_2657593 |
| NKG2D | PE | 320806; BioLegend | 1D11 | AB_492960 |
| CD69 | PE-CF594 | 562617; BD | FN50 | AB_2737680 |
| TIM3 | BV786 | 742857, BD | 7D3 | AB_2741100 |
| CD337/NKp30 | PE-daz594 | 325232, Biolegend | P30-15 | AB_2814185 |
| CD226/DNAM-1 | BV510 | 338330, Biolegend | 11A8 | AB_2728300 |
| CD159a/NKG2A | FITC | 130-113-565, Miltenyi | REA110 | AB_2733623 |
| CD45 | AF700 | 560510, BD | 30-F11 | AB_1645208 |
| CD159a/NKG2A | BUV737 | 749682, Thermofisher | 131411 | AB_2873939 |
| CD159c/NKG2C | BV650 | 748165, Thermofisher | 134591 | AB_2872626 |
| CD96 | BB515 | 564774, BD | 6F9 | AB_2738945 |
| CD200R | PE | 12-9201-42, Thermofisher | OX108 |  |
| HLA-DR | PE-Fire 810 | 307683 Biolegend | L243 | AB_2904336 |
| CD253/TRAIL | APC | 308210, Biolegend | RIK-2 | AB_2564398 |
| TIGIT | AF647 | 372724, Biolegend | A15153G | AB_2715972 |
| CD38 | APC-Fire 810 | 303550, biolegend | HIT2 | AB_2860784 |
| CD56 | cFluor R720 | SKU R7-20089, Cytek | 5.1H11 |  |
| CD94 | APC-Fire 750 | 305518, Biolegend | DX22 | AB_2734275 |
